# Live birth in a woman without ovaries after autograft of frozen-thawed ovarian tissue combined with growth factors

**DOI:** 10.1186/1757-2215-6-33

**Published:** 2013-05-07

**Authors:** Justo Callejo, Cristina Salvador, Santiago González-Nuñez, Laura Almeida, Luciano Rodriguez, Laura Marqués, Ana Valls, José Maria Lailla

**Affiliations:** 1 Department of Obstetrics and Gynecology, Hospital Sant Joan de Déu, Faculty of Medicine-University of Barcelona, Barcelona, Spain; 2 Tissues Bank Division, Banc de Sang i Teixits, Barcelona, Spain; 3 Center for Human Reproduction, Clínica Sagrada Familia, Barcelona, Spain; 4 Hormone Laboratory, Hospital Sant Joan de Déu, Barcelona, Spain

**Keywords:** Platelet rich plasma, PRP, Antimüllerian hormone, AMH, Cryopreservation, Fertility preservation, Ovarian tissue transplantation

## Abstract

Currently, cryopreservation of oocytes, embryos and ovarian tissue is considered the basis of fertility preservation programs for women with cancer and other diseases who are rendered sterile by gonadotoxic drugs or radiation.

Numerous studies have confirmed that autograft of frozen-thawed ovarian tissue can restore ovarian function and fertility. A total of twenty-two live births have been reported but we still have to consider this technique as experimental. The main problem is that the implant undergoes ischemia until neoangiogenesis is restored, resulting in significant follicular loss.

At the moment, there are numerous publications in different medical fields that publish successful experiences with plasma rich in platelets (PRP) in different clinical situations promoting angiogenesis. Thus, we considered the possibility of using it in the field of ovarian autologous transplantation in order to improve the vascularization of the implant and its quality. For this, both thawed ovarian tissue as practiced pockets on the rear side of the broad ligament which have been placed, have been impregnated with PRP. We can say that the implant treated in this way has had a rapid and successful response.

We report a special interesting case because this is the first time that this technique is performed successfully in a woman without ovaries combined with growth factors to promote neoangiogenesis. Obviously, the results of the hormonal response come exclusively from the implanted tissue in these special conditions.

## Background

Currently, cryopreservation of oocytes, embryos and ovarian tissue is considered the basis of fertility preservation programs for women with cancer and other diseases who are rendered sterile by gonadotoxic drugs or radiation.

Numerous studies have confirmed that autograft of frozen-thawed ovarian tissue can restore ovarian function and fertility. A total of twenty-two live births in twelve women have been reported [1-3], but this technique is still considered experimental. The main problem is that the implant undergoes ischemia until neoangiogenesis is restored, resulting in significant follicular loss as noted in some original articles [4-6].

We report a special interesting case because this is the first time that this technique is performed successfully in a woman without ovaries combined with growth factors to promote neoangiogenesis. Obviously, the hormonal response comes exclusively from the implanted tissue under these special conditions.

## Case presentation

In 2001, when the patient was 20 years old, she underwent an urgent left oophorectomy due to a twisted necrosed dermoid cyst of 15×10 cm. After two months, in the postsurgical control, the ultrasound examination showed another cyst of 3x2cm in the right ovary that again suggested a dermoid cyst. A new surgery was proposed to the patient with a conservative intention but after the cystectomy only 25% of healthy ovarian tissue was left and it was emplaced opposite to the vascular hilum and connected to it by just a fibrous band with poor vascularization. Therefore, we proceeded to resect and cryopreserve the remained tissue according to the fertility preservation protocol in oncological patient of our hospital. The patient was informed of this eventuality. The anathomo-pathologycal analysis confirmed the clinical diagnosis of dermoid cyst in both tumors.

Clinical and biochemical premature ovarian insufficiency (POI) was immediately observed; therefore, hormonal replacement treatment (HRT) was started.

In 2011, when she was 30 years old, the patient wished to become pregnant. HRT was stopped and the cryopreserved ovarian tissue was reimplanted using growth factors to boost neoangiogenesis. Four-and-a half months later, the ovarian function was restored and the first spontaneous menstruation ensued. Then, on the third day of menstrual bleeding, ovarian stimulation for in vitro fertilization/intracytoplasmatic sperm injection (IVF/ICSI) was started. In each implant one follicle 6 mm developed. Peripheral hormones showed the following levels: follicular stimulating hormone (FSH) 7,9 mIU/ml; and estradiol 58 pg/ml. We used 300 units of recombinant FSH daily and on the third day we added 150 units of recombinant luteinizing hormone (LH), also daily (Gonal-F and Luveris, Merk Serono). On day 6 of the stimulation, a daily administration of 0.25 mg of a gonadotropin releasing hormone (GnRH) antagonist (Cetrotide, Merck Serono) was initiated. On the ninth day of stimulation egg retrieval was performed. Estradiol levels were 316 pg/ml. The ovulation was triggered with human chorionic gonadotropin (HCG) 250 mcg (Ovitrelle, Merck Serono) followed by vaginal administration of 200 mg micronized progesterone 3 times a day (Utrogestan, Laboratorios SEID S.A.) and estradiol hemihydrate 0.78 mg transdermal every three days (Estradot, Novartis Farmaceutica). The endometrial thickening was 10 mm, and 3 follicles were retrieved (two 14 mm on the right side and one 11 mm on the left side). Two oocytes were obtained, one metaphase II (MII) and the other metaphase I (MI) that developed to MII after 7 hours of culture). Both were inseminated by ICSI. On the day +2 we transferred a 4 regular mononucleated cells embryo and the other with 15% of fragmentation. Serum testing for HCG was positive 13 days after the embryo transfer. Repeated ultrasonography during the pregnancy showed normal fetal growth and development. At 38 weeks and 6 days of gestation, a healthy boy weighing 3.500 Kg, Apgar 9–10, was delivered by caesarean section on July 31, 2012.

### Freezing and thawing of ovarian tissue

Ovarian tissue was frozen in pieces of 2–3 mm^3^ using dimethylsulphoxide (DMSO) and thawed according to the protocol described elsewhere by our group [7].

### Platelet rich plasma (PRP)

Platelet rich plasma was prepared starting with 60 mL of patient´s blood drawn into tubes containing 3.2% of citratated dextrose. Blood was centrifuged at 280 g for 15 minutes at room temperature, plasmatic fraction was isolated and a second centrifugation was performed at 680 g during 20 minutes. The platelet pellet was diluted in 5 mL of autologous plasma. Finally, the ovarian tissue was coated with PRP before implantation.

Multiple preparations of PRP for clinical use have been described. Differences in volume, concentration of platelets, white blood cells and even in the final presentation (liquid or gel) define the observed variability of those products. We performed a double-spin centrifugation protocol to obtain the highest recovery of platelets to promote a rapid neovascularization of the graft [8,9] but with the minimal concentration of blood cells to avoid undesirable catabolic and inflammatory related events that could potentially affect the ovarian tissue [10,11].

### Surgery

**Figure 1:**
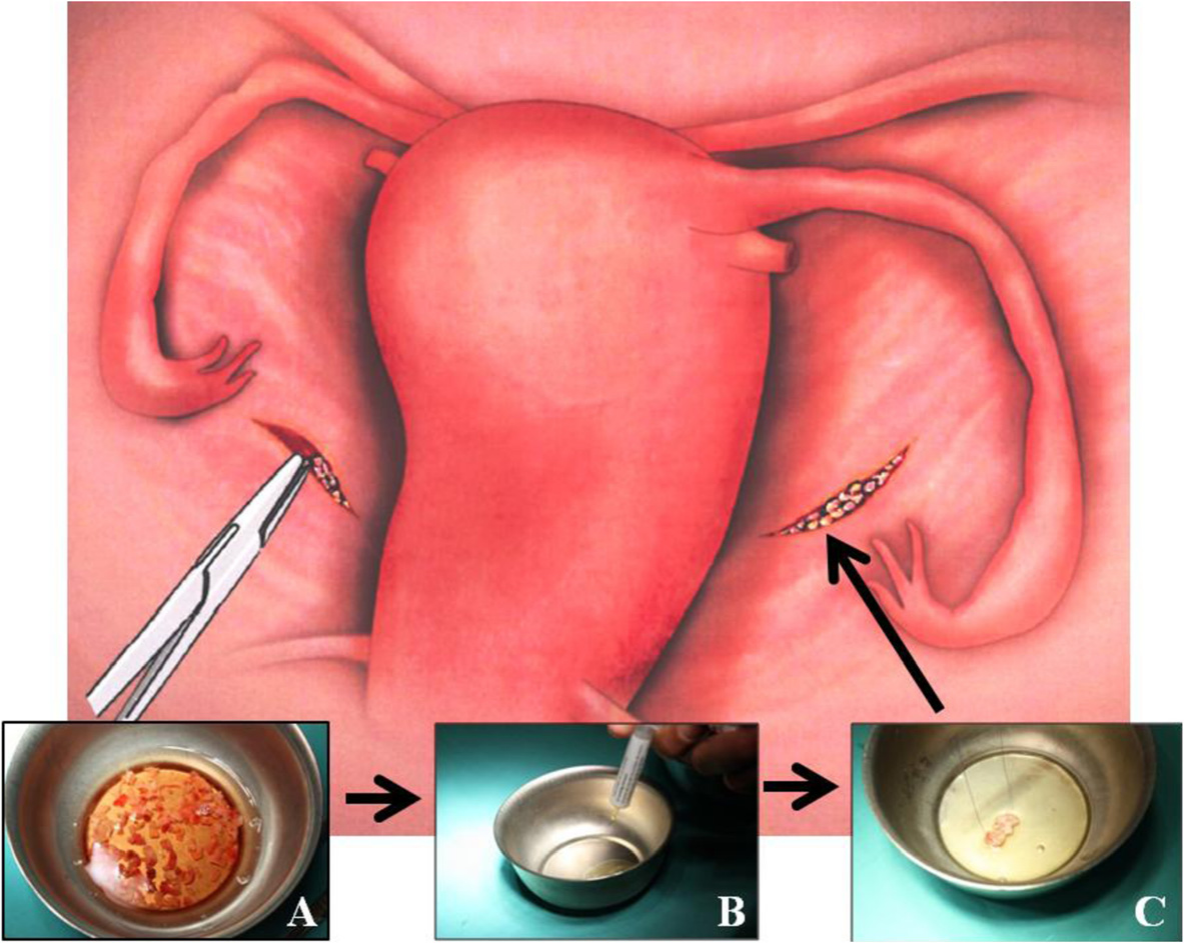
**The surgical procedure is extremely easy.** The thawed ovarian tissue (**A**) is impregnated in a gel preparation of PRP (**B**) and it is transferred using laparoscopic surgery to the pockets located in the posterior surface of the broad ligaments (**C**).

For the reimplantation we used all the frozen-thawed small cubes of ovarian tissue (1–2 mm^3^). We created a peritoneal pocket in both posterior surfaces of the broad ligament by laparoscopic surgery, where we placed the cubes. Previously, we used a gel preparation of PRP to impregnate the cubes and, after they have been located in the peritoneal pockets performed, it proceeds to fill these with the platelet gel using the 5 mL supplied (Figure 1).

### Hormone laboratory

Serum FSH, LH and HCG were measured by a chemiluminiscent microparticle immunoassay in an ARCHITECT analyzer (Abbott Laboratories, Abbott Park, IL). Progesterone was measured by a Chemiluminescent enzyme immunoassay in a IMMULITE2000 analyzer, SIEMENS Healthcare Diagnostics. Anti-Mullerian Hormone (AMH) serum was measured using an ultrasensitive two-site ELISA (BECHMAN COULTER, USA). Serum inhibin B concentrations were measured using a highly sensitive two-site enzymelinked immunosorbent assay ELISA; (Diagnostic Systems Laboratories, Inc).

### Ethical considerations

The protocol was approved by the local Ethical Committee and the administrative authorization Ref. H08000875 Departament de Salut (Generalitat de Catalunya). The patient and her partner received written information about all the process and signed the informed consent.

## Results

**Figure 2:**
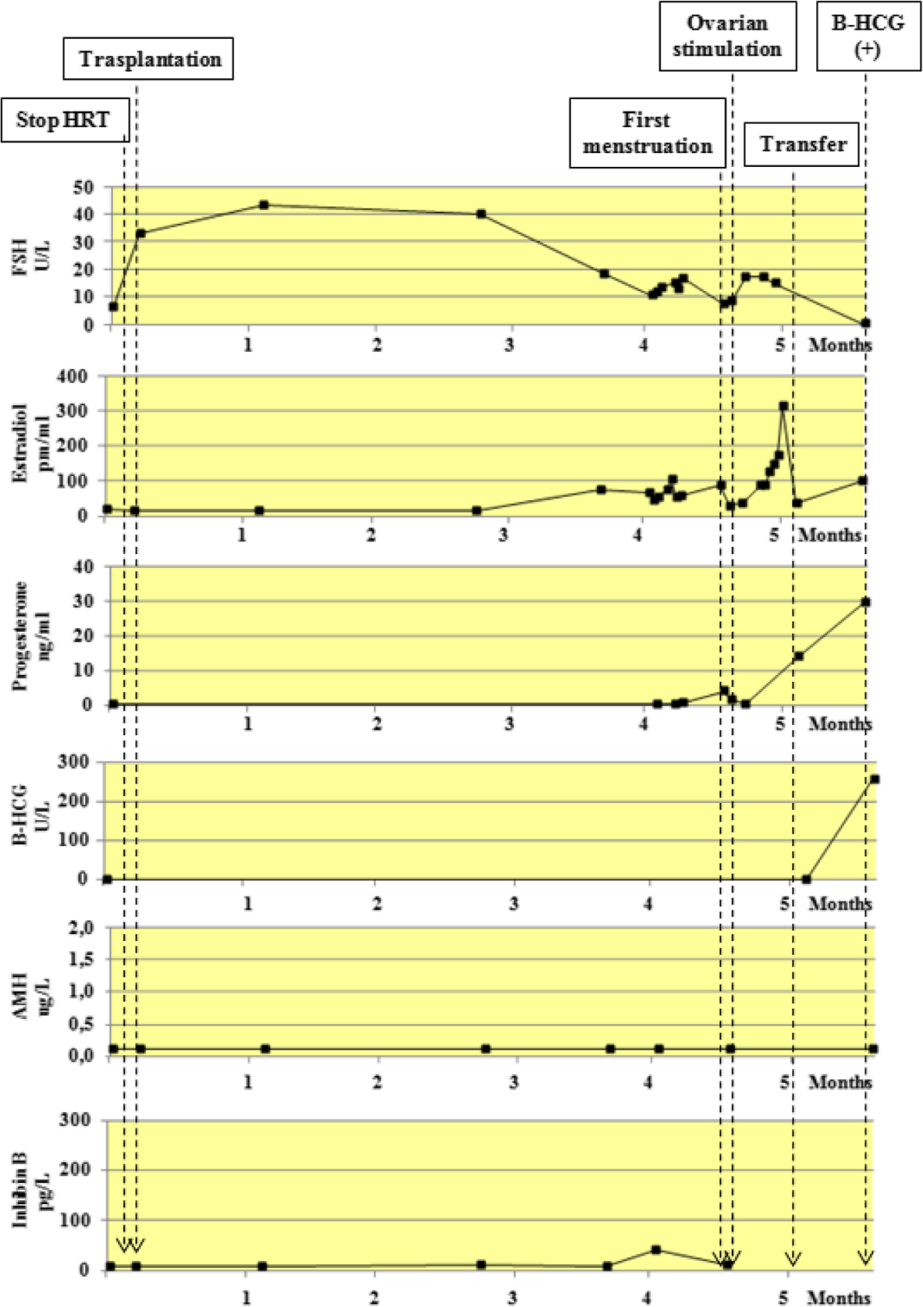
Peripheral hormones levels of FSH, estradiol, Progesterone, HCG, AMH and Inhibin B, from the first menstruation until the first positive HCG determination.

**Figure 3:**
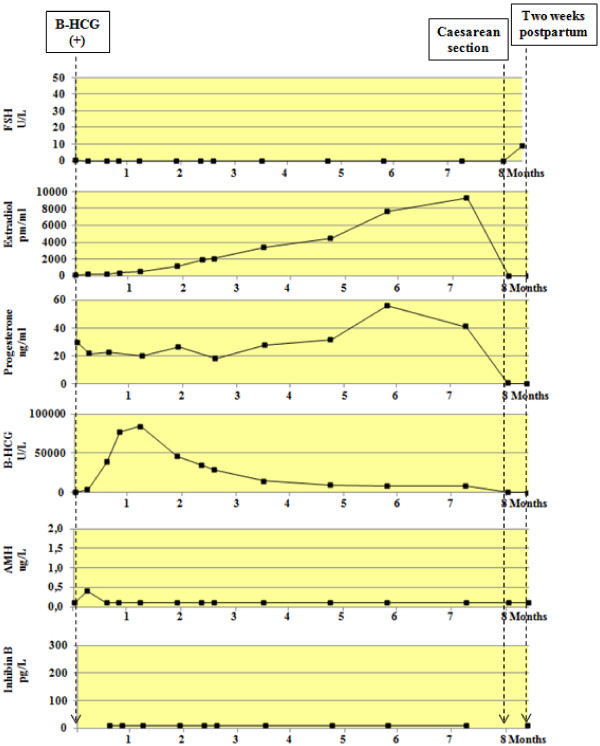
Peripheral hormones levels of FSH, estradiol, Progesterone, HCG, AMH and Inhibin B, during pregnancy and immediate pospartum.

Figure 2 shows the peripheral hormones levels of FSH, estradiol, Progesterone, HCG, AMH and Inhibin B, from the first menstruation until the first positive HCG determination. Figure 3 shows the hormonal changes during pregnancy and immediate postpartum.

## Discussion

Certainly, the main problem of autograft of frozen-thawed ovarian tissue is the loss of more than 50% of follicles caused by the ischemia that the autograft suffers until neoangiogenesis is restored [4-6]. Today, investigators are working to improve this problem.

PRP is a blood product where a high level of platelets is concentrated (about 1.000.000 of platelets/μL in 5 ml of plasma) but with growth factors concentration 3 to 5 times greater than plasma. Growth factors are stored in α granules, and include platelet derivated growth factor (PDGFs), Transforming growth factor-beta (TGF-β), vessel endothelial growth factor (VEGF), epidermal growth factor (EGF), fibroblast growth factor (FGF) e insuline-like growth factor (IGF) and some others [12,13]. These cytokines play an important role in cellular proliferation, chemotaxis and differentiation of mesenchymal and other cells and promote angiogenesis [13].

There are already numerous publications in different medical fields reporting successful experiences with PRP that improve neoangiogenesis in different clinical situations [8-16]. Thus, we considered the possibility of using it in the field of ovarian autologous transplantation in order to improve the vascularization of the implant and its quality.

The result has been successful in obtaining a healthy baby in the first cycle of stimulation, but it has been seen before [17,18] without the use of PRP. But with a single case, one cannot draw any conclusions. More studies are required to determine whether the use of PRP represents a contribution.

We would like to highlight the results of the levels of AMH: throughout the process, peripheral levels of this hormone were undetectable (<0.1 ng / ml). These results are consistent with the series of Janse et al. [19] and Grave et al. [20]. Very low or undetectable levels of AMH at the time of conception indicate a poor and irregular pool of them which have already started their development from primordial follicles.

We would like to make some observations on the indication of egg retrieval when follicle sizes are about 14–15 millimeters. Usually, these can be considered immature follicles, moreover if we obtain levels of estradiol that correspond to a mature follicle in transplanted patients with this follicular size (14–15 mm), we should consider that indirect clinical criteria of follicular maturity in the autograft (follicular size and rate of estradiol), do not necessarily have to be the same as in the normal inserted ovary. When we perform ultrasound measurements of the follicles we are measuring the antral diameter without considering the possibility of a granulosa layer hyperplasia that provides estradiol characteristic levels of a mature follicle. In the experimental model, when the reimplanted ovarian tissue is functioning, our group shows this possibility [21,22].

At this point we would like to draw attention on the recommendations of Oktay et al. in 2004 [23] who proposed follicular maturity at a size of 10–11 mm in this type of tissue. Demesteere et al. [24] who reported a spontaneous pregnancy after orthotopic graft in 2006, explains that the follicles which developed in the subcutaneous implant never reached the sufficient size to be retrieved (maximum 13 mm). A year later, the same author [25], in the second reimplantation to the same patient, which fortunately concluded with the announcement of the third live birth from cryopreserved ovarian tissue, announced that the two retrieved follicles of the implanted ovarian measured 15 mm at the time of ovulation. Fourteen days later chorionic gonadotropin levels were detected.

Nevertheless, many authors have retrieved follicles above 17 mm obtaining good quality MII oocytes [1] and these arguments must consider only speculation.

## Conclusion

We report the second pregnancy occurred after ovarian tissue cryopreservation for benign ovarian pathology after bilateral oophorectomy. Growth factors are used in combination with frozen-thawed ovarian tissue to boost neoangiogenesis. The result of this case was successful. However, more studies are required to determine whether the use of PRP represents a contribution.

### Consent

Written informed consent was obtained from the patient for publication of this Case Report and any accompanying images. A copy of the written consent is available for review by the Editor-in-Chief of this journal.

## Abbreviations

PRP: Platelet rich plasma; AMH: Antimullerian hormone; POI: Premature ovarian insufficiency; HRT: Hormonal replacement treatment; IVF: in vitro fertilization; ICSI: Intracytoplasmic sperm injection; FSH: Follicle stimulating hormone; LH: Luteinizing hormone; GnRH: Gonadotropin-releasing hormone; HCG: Human chorionic gonadotropin; MII: Metaphase II; MI: Metaphase I; DMSO: Dimethyl sulfoxide; PDGF: Platelet derivate growth factor; TGF-β: Transforming growth factor-beta; VEGF: Vessel endothelial growth factor; EGF: Epidermal growth factor; FGF: Fibroblast growth factor; IGF: Insuline-like growth factor.

## Competing interests

The authors declare that they have no competing interests.

## Authors’ contributions

JC has conceived the study, participated in its design and coordination and he has drafted the manuscript; CS has been involved in drafting the manuscript and revising it critically for important intellectual content; SG-N has been performed the surgical intervention; LA has participated in the design of the study and drafted the manuscript; LR has carried the preparation of PRP and drafted the manuscript; LM has carried out laboratory the work in the human reproduction laboratory; AV has carried out the hormone assays and drafted the manuscript, and JML has given final approval of the version to be published. All authors have read and approved the final manuscript.

## Authors’ information

From Department of Obstetrics and Gynecology, Hospital Sant Joan de Déu; Faculty of Medicine-University of Barcelona, Barcelona, Spain (J.C., C.S., S.G.N., L.A. J.M.L.); Tissues Bank Division, Banc de Sang i Teixits, Barcelona, Spain (L.R.); Center for Human Reproduction, Clínica Sagrada Familia, Barcelona, Spain (L.M.) and Hormone Laboratory, Hospital Sant Joan de Déu, Barcelona, Spain (A.V.)
